# SureChEMBL: a large-scale, chemically annotated patent document database

**DOI:** 10.1093/nar/gkv1253

**Published:** 2015-11-17

**Authors:** George Papadatos, Mark Davies, Nathan Dedman, Jon Chambers, Anna Gaulton, James Siddle, Richard Koks, Sean A. Irvine, Joe Pettersson, Nicko Goncharoff, Anne Hersey, John P. Overington

**Affiliations:** 1 European Molecular Biology Laboratory, European Bioinformatics Institute (EMBL-EBI), Wellcome Genome Campus, Hinxton, Cambridgeshire CB10 1SD, UK; 2 Digital Science, London N1 9XW, UK; 3 NetValue Ltd, Hamilton 3240, New Zealand; 4 McKinsey & Company, London SW1Y 4UH, UK

## Abstract

SureChEMBL is a publicly available large-scale resource containing compounds extracted from the full text, images and attachments of patent documents. The data are extracted from the patent literature according to an automated text and image-mining pipeline on a daily basis. SureChEMBL provides access to a previously unavailable, open and timely set of annotated compound-patent associations, complemented with sophisticated combined structure and keyword-based search capabilities against the compound repository and patent document corpus; given the wealth of knowledge hidden in patent documents, analysis of SureChEMBL data has immediate applications in drug discovery, medicinal chemistry and other commercial areas of chemical science. Currently, the database contains 17 million compounds extracted from 14 million patent documents. Access is available through a dedicated web-based interface and data downloads at: https://www.surechembl.org/.

## INTRODUCTION

Undoubtedly, the continued growth in the number of published patent applications worldwide and the use of sophisticated automated text, image and data mining have opened new opportunities and challenges in the field of patent information management and searching (1). This is true especially in the context of chemical and pharmaceutical patents, where useful and accurate chemical annotation and indexing poses notable challenges (1,2).

Viewed as technical documents, patents are a valuable and unique source of information in scientific research. It is estimated that only a small fraction of the new science and technology first reported in patents is subsequently disclosed in scientific literature sources (3). Moreover, the first public disclosure of new chemical entities, scaffolds and series typically takes place in patent applications prior to their publication in scientific journals. In addition to novel composition of matter, chemical patent documents contain information on reactions and synthetic pathways (4,5), biomarkers, assays, experimental conditions, active ingredients and catalysts, as well biological targets (6), mechanisms of action, bioactivity data and disease indications. For example, through searching the patent corpus, a scientist interested in a particular protein target may be able to identify key compounds active against that target (possibly with quantitative bioactivity measurements) that are not present in other sources, such as commercial or publicly available bioactivity databases or peer-reviewed scientific literature (7,8). Similarly, large-scale mining of the patent corpus may identify potential associations between targets and diseases or targets and chemical scaffolds that would otherwise not be apparent from isolated analysis of single, or few, documents.

Finally, patents are a primary source for competitive industrial and academic intelligence, citation analysis and research productivity metrics (9). Retrieval of applicant data, for example, can give an overview of which companies are working in a particular area (e.g. target or indication) in advance of these programmes reaching clinical trials.

Major patent authorities, such as the European Patent Office (EPO, https://www.epo.org), the United States Patent and Trademark Office (USPTO, http://www.uspto.gov), the Japanese Patent Office (JPO, https://www.jpo.go.jp) and the World Intellectual Property Organization (WIPO, http://www.wipo.int) provide web resources which can be accessed and queried online. In addition, the patent documents are made available usually as XML, HTML or as text/image PDFs. However, these resources lack the systematic and continuous chemical annotation and full-text searching capabilities. Given the strong financial incentives and interest in patent chemistry extraction and searching, related products in this field have been traditionally closed and commercial. Databases such as CAS SciFinder (https://scifinder.cas.org), Elsevier Reaxys (http://www.elsevier.com/solutions/reaxys) and Thomson Reuters Pharma (http://lifesciences.thomsonreuters.com) are the standard tools used almost solely in industry, typically due to significant cost. Moreover, given that the relevant content is usually manually extracted from the patents, compromises have to be made with regard to the coverage and turnaround time due to the sheer volume of published patent documents from patent authorities worldwide.

More recently, publicly available resources containing chemistry extracted from patents have emerged. As opposed to manual curation, these are based on large-scale extraction of chemical content in an automated fashion by means of text, image and file mining, followed by name-to-structure and image-to-structure conversion. SCRIPDB (10) is a chemical structure database containing compounds and reactions extracted from the Complex Work Unit (CWU) files of granted US patents (http://www.uspto.gov/patent/initiatives/complex-work-unit-pilot-program). Since 2001, the USPTO requires applicants to submit chemical structures and reactions depicted in their patent applications as MDL molfiles and ChemDraw CDX binary files. These files are then automatically processed by the SCRIPDB pipeline. In terms of coverage, SCRIPDB contains more than 10 million distinct chemical structures from more than 100 000 granted patent documents published between 2001 and 2011. The interface provides the standard chemical searching functionality against the compound repository and external links to Google Patents (https://patents.google.com) are provided for the search hits. While SCRIPDB was one of the first freely available primary sources of patent chemistry, it is limited to CWU files coming from granted US patents. US patent applications and other patent authorities, as well as additional important sources of chemical entities, such as the full text, are excluded from the SCRIPDB pipeline. Importantly, the resource has not been updated with new patents and compounds since 2011.

The ChEBI database (11,12) also contains chemistry automatically extracted from the title and abstract of a subset of biologically relevant patent documents; the project was carried out in collaboration with the EPO and the SLING consortium (11). The compounds are searchable on the ChEBI interface and there are links to EPO's Espacenet website (http://worldwide.espacenet.com) with bibliographic patent data for the search hits. Similarly to the SCRIPDB resource, ChEBI is limited in coverage to a subset of EPO patents, which has not been updated in the last years.

Another publicly available source following the automated patent mining paradigm is the dataset contributed by IBM to the US National Institutes of Health (NIH) (IBM press release: http://www-03.ibm.com/press/us/en/pressrelease/36180.wss). The data were extracted using IBM's Strategic IP Insight Platform (SIIP) (http://www-935.ibm.com/services/us/gbs/bao/siip). The first release of the data contained more than 2.3 million chemical structures extracted from about 4.7 million full-text patents (EPO, WIPO and USPTO), as well as 11 million biomedical journal abstracts from 1976 to 2000. For this period, images in patents were not processed and chemical structures were only derived from text. Subsequently, the coverage was extended to include patents published up to the end of 2010 and chemical structures were additionally derived from US CWUs and images for the period 2001 to 2010. The resource consists of several flat files with detailed patent-compound association information. The files are fully integrated and searchable in PubChem (13) and UniChem (14). While the SIIP dataset is much broader in scope and coverage compared to SCRIPDB and ChEBI, it is static, and lacks any chemical data from the past 5 years.

In addition to compounds, significant efforts have been made towards the automated extraction of chemical reactions and associated conditions and yields from patent documents. Recently, more than one million reactions extracted from US patent documents were released in the public domain by NextMoveSoftware (press release: https://nextmovesoftware.com/blog/2014/02/27/unleashing-over-a-million-reactions-into-the-wild). The reactions were extracted by means of automated text mining of the relevant experimental sections reported in patents, covering the period between 1976 and 2013.

Finally, there are secondary public resources that integrate several sources of patent chemistry, such as PubChem (13) and UniChem (14). The former contains compounds and links to patents from several major depositors, such as IBM, ChEBI/EPO, SCRIPDB and Thomson Pharma. UniChem also provides access to all PubChem integrated patent-related compound sources mentioned above and allow for exact and connectivity searches via its interface and web services (14,15).

Most of the publicly available primary patent chemistry sources described above are rather limited in scope and, crucially, their coverage is not up to date with the weekly new patent output. They largely offer links from compounds to patents and, to a lesser extent, *vice* *versa*. However, there is no combined keyword- and chemistry-based patent searching, detailed compound to patent section mapping, or refined data exporting functionality. SureChEMBL bridges these gaps by offering continuous, up-to-date and broad chemical annotation and curation of the extracted chemistry from patent documents by means of automated full-text, image and attachment file mining. This is complemented by advanced patent text and bibliographic metadata querying and chemistry searching functionality available on its dedicated web interface. Finally, there are several options for exporting compound and patent associations and accessing the data in batch. To our knowledge, this is the first time a patent chemistry database of this scale and scope is freely available as a public resource.

SureChEMBL was derived from a commercial chemistry patent mining product originally developed as SureChem by Digital Science Ltd. In late 2013, the system was donated to the EMBL-EBI with the remit to expose full functionality and underlying chemical structure content to the public domain (EMBL-EBI press release: http://www.ebi.ac.uk/about/news/press-releases/SureChEMBL). Following a short migration period, this objective was achieved and since then EMBL-EBI has committed to provide an on-going service and support further development of the resource.

## DATA CONTENT

### Data extraction pipeline

**Figure 1.**
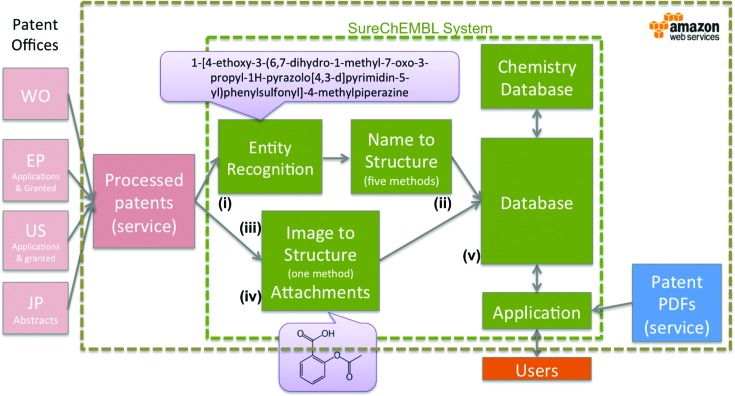
Overview of the SureChEMBL data pipeline from the raw patent feed to the standardized compounds in the database.

The SureChEMBL data pipeline is summarized schematically in Figure 1. It consists of several steps, namely (i) chemical entity recognition, (ii) name to structure conversion, (iii) image to structure conversion, (iv) attachment CWU mol file parsing and (v) compound standardization and registration.

The patent feed from four major patent authorities, including the full text from USPTO, EPO, WIPO and titles and abstracts from the JPO, is aggregated, digitized where applicable and processed in XML format by a third party patent content vendor, namely IFI Claims. Then, an optimized chemical entity recognition algorithm (developed by SureChem) tokenizes and scans the full text of a patent (i.e. all four sections, title, abstract, claims and description) for named chemical entities (e.g. systematic, semi-systematic, common and drug non-proprietary and trade names). The entity recognition algorithm consists of a dictionary, and a chemical name classifier, trained on a representative set of systematic names. In addition, the algorithm uses a chemical name grammar, featuring a set of rules to identify and parse complex chemical names in text. After the identification and extraction of the chemical entities, these are then submitted to five different name-to-structure tools, namely ACD/Labs, ChemAxon, OpenEye, PerkinElmer (16) and OPSIN (17). These attempt to convert a named entity to the corresponding structure or structures. If the conversion fails initially, there are iterative steps in place, including CaffeineFix (NextMove Software (18)). These steps attempt to fix spelling mistakes and potential optical character recognition errors in the chemical name by trying a number of single and multiple character replacements (e.g. converting a spurious ‘1’ to ‘l’, as differentiating between these two characters can be problematic). The successfully converted structures (up to five different ones for an input entity) are then standardized according to a set of rules; for example, explicit hydrogens are suppressed, fragments are removed and functional groups, such as nitro groups, are normalized. The resulting compounds are registered (if new) to a chemically aware relational database (ChemAxon JChem). At the same time, in a different branch of the pipeline, the images attached to a patent document are submitted to an image-to-structure converter (Keymodule CLiDE (19)) and the successfully converted structures are similarly processed and stored in the chemical database. In addition to the images, the pipeline also processes chemical structure drawings encoded as molfile (.mol) attachments which are provided by the USPTO. Finally, the full querying and data exporting functionality is exposed to the end users via a dedicated web interface (www.surechembl.org).

The whole pipeline is comprised of several virtual machines which communicate with each other via a message queue system and are hosted in the Amazon Web Services (AWS) cloud. Importantly, the SureChEMBL pipeline is dynamic and fully automated in nature: every week there are new applications and granted patent documents published by their respective authorities and these are subjected to the same chemical annotation and extraction procedure without manual intervention or required curation.

### Data coverage

The SureChEMBL database is not limited to chemically annotated or chemically relevant patent documents. Instead, it contains at least the bibliographic information (title, abstract, publication year, etc.) for patents published from 1920 onwards by more than 90 countries (Table 1). The data coverage for the chemically annotated patent corpus includes full text (for USPTO, WIPO and EPO) and bibliographic information (for JPO) for both applications and granted patents with the earliest annotated document published in 1976. Both annotated and unannotated patents are fully searchable and accessible on the SureChEMBL interface, as described later. With regard to structure extraction from images and attachment CWUs, this is available for patents published in 2007 onwards.

**Table 1.** The patent content and coverage of the SureChEMBL database

	Data	Description and languages	Years
EP applications	Bib. data	DocDB + Original	From 1978
	Full text	Original (EN, DE, FR)	
EP granted	Bib. data	DocDB + Original	From 1980
	Full text	Original (EN, DE, FR)	
WO applications	Bib. data	DocDB + Original	From 1978
	Full text	Original (EN, DE, FR, ES, RU)	From 1978
US applications	Bib. data	DocDB + Original	From 2001
	Full text	Original (EN)	From 2001
US granted	Bib. data	DocDB + Original	From 1920
	Full text	Original (EN)	From 1976
JP applications	Bib. data	DocDB	From 1973
		English abstracts/titles	From 1976
JP granted	Bib. data	DocDB	From 1994
90+ countries	Bib. data	DocDB	From 1920

It is worth mentioning that the WIPO does not grant patents as this a prerogative of the national or regional patent authorities. DocDB refers to a database provided by the EPO, containing comprehensive bibliographic information for patent documents released worldwide.

### Data model

The most important entity types within SureChEMBL are the patent documents (from which the chemical annotations are extracted), the chemical named entities (the chemistry-related mentions in the text) and compounds (which are converted from chemical entities). For each annotated patent document, the locations in text in which chemical entities were identified are recorded along with the chemical entity identifier. In case of images and molfile attachments, a second table holds the source and chemical entity information. A dictionary of unique chemical entities is in turn mapped to one or more compounds though the name and image-to-structure tools. A dictionary of standardized compounds is maintained along with additional representations and properties, such as connection table, standard InChI and InChI Key (20), molecular weight, calculated logP and global frequency, i.e. how many times the compound is seen in the entirety of the SureChEMBL chemically annotated patent corpus. Compound uniqueness and allocation of new identifiers is handled by ChemAxon JChem during registration to the database. In practice, canonical SMILES strings (21) generated by the ChemAxon Marvin toolkit define compound uniqueness. Thus, different stereoisomers and tautomers of the same structure will be given different identifiers. Each entry of the compound dictionary is assigned a unique SureChEMBL identifier, which takes the form of a ‘SCHEMBL’ prefix followed immediately by an integer (e.g. SCHEMBL1353 is the compound 2-(acetyloxy)benzoic acid, also known as aspirin). In addition, patent documents have a unique external identifier, called SureChEMBL Patent Number (SCPN), of the format CC-n-KK, where CC is the 2-digit country code, *n* is the patent number and KK is the 1- or 2-digit kind code. This format is consistent with the internal identifiers used by the patent content provider. An example of an SCPN is WO-2012088469-A1, referring to a patent application from the WIPO. The full specification of the patent number format is available on the SureChEMBL interface.

### Current data content

At the moment of writing, the SureChEMBL database contains more than 17 million distinct compounds extracted from more than 14 million patent documents, spanning a time range from 1970 to present. Given that SureChEMBL is a live, dynamically updated resource, what is perhaps even more impressive is the rate at which new compounds and annotated patent documents are added to the database every month, which is ∼80 000 and 50 000, respectively. The daily patent feed coupled with the fully automated pipeline allow for rapid annotation and indexing of chemical structures, extracted from new patent documents. In most cases, the turnaround time from the publication of a patent by an authority to the patent and its associated compounds being accessible and searchable on the SureChEMBL interface is between 1 and 4 days.

### Data exchange and cross-references

SureChEMBL compound data is available via UniChem (14,15), a freely available, large-scale compound cross-referencing service based on the InChI representation (20). UniChem provides the full mapping of the SureChEMBL compounds against the rest of the 27 compound sources currently available, and is used to provide dynamic cross references between SureChEMBL and the ChEMBL database (22,23), also hosted at EMBL-EBI. Moreover, UniChem provides RESTful web services that allow for quick structure look-ups against the SureChEMBL compound repository. Finally, full Oracle dumps (including the SureChEMBL compound source) of UniChem are also available to download via ftp. UniChem is updated with new compounds extracted by SureChEMBL on a weekly basis.

SureChEMBL is also a fully integrated data source in the PubChem database. Incremental updates of new compounds are added quarterly. Finally, cross-references to SureChEMBL patent documents are provided by the Europe PMC interface (24) for the subset of the patent corpus (titles and abstracts) available there.

## DATA ACCESS

### The SureChEMBL interface

**Figure 2.**
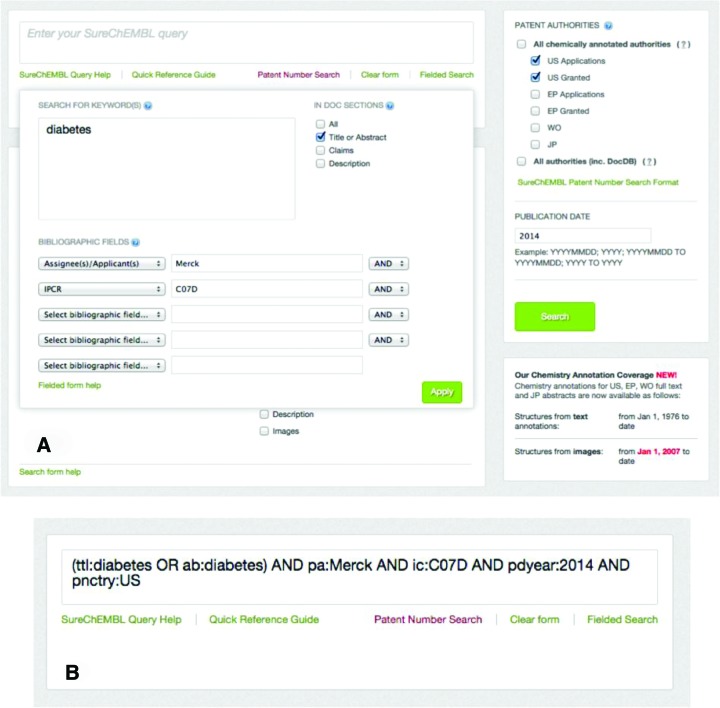
(**A**) Field keyword-based search against full text and patent bibliographic metadata. (**B**) The equivalent search using the Lucene query fields syntax.

**Figure 3.**
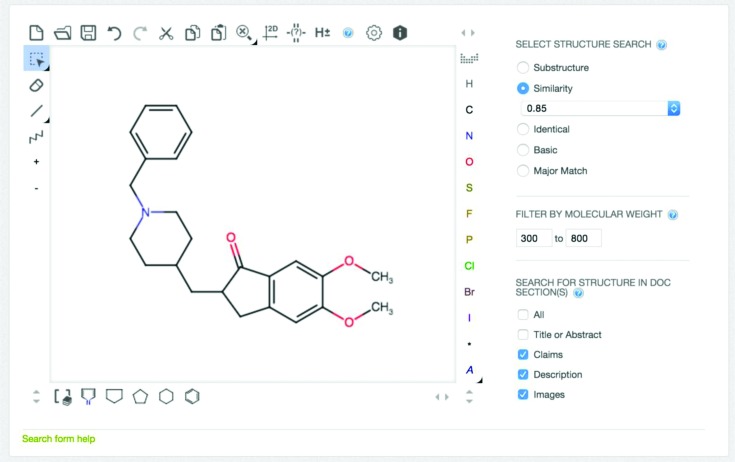
Similarity search for the near neighbours of the approved drug *donepezil*. The search results will have a molecular weight range of 300–800. Furthermore, only compounds that are extracted from the claims or description sections and images will be retrieved.

The SureChEMBL database is accessible via a simple, user-friendly interface at: https://www.surechembl.org. This interface allows users to search for compounds and patent documents of interest with a very high degree of granularity, as well as filter and retrieve the desired subset of results. More specifically, the interface has the following querying capabilities:

- Keyword-based searching against the full text of patent document, including title, abstract, claims and description sections. This is complemented by keyword-based searching against bibliographic and other metadata, such as patent identifier, patent authority, kind code (i.e. a code denoting the status of a patent, for example application or granted), applicant/assignee information, publication year, inventor, patent classification code (i.e. a manually assigned hierarchical classification system based on the contents of a patent (25)), priority date and country (i.e. the date and patent authority of the first application filing), patent language, etc. All keyword-based searches are powered by a Lucene index backend, which is updated nightly. The interface also provides two alternative ways to input the query, either using the field search modal window or as a combination of Lucene query fields in the main search box (Figure 2). Keywords can be combined with Boolean operators (AND, OR, NOT) and nested in parentheses, to allow for very granular queries. Finally, more advanced querying features, such as wildcards, and exact and proximity searches are also supported. Since only compounds are currently extracted from SureChEMBL patent documents, the keyword search provides a mechanism by which users can search for patents relating to other entities of interest such as targets, diseases or biological processes.
- Searching using a chemical structure or substructure as query. Similarity search at a given Tanimoto threshold and substructure search of the compound database are both supported and are implemented using the ChemAxon indexing technology. The query can be drawn using the javascript-based drawing tool available. Alternatively, the query (sub)structure can be pasted as a SMILES, molfile or SMARTS string (21); finally, input name to structure conversion is also supported to initiate a structure search. Additional faceting of the search results is also provided by indicating the specific source of the compounds (section of patent, images or molfiles) (Figure 3). This additional granularity allows users to specify the exact location the compounds were extracted from during a chemistry search, e.g. claims or description and thus focus only on the relevant, novel compounds of interest.
- A combination of both a keyword-based and a chemistry-based query. For example, a user may search for compounds that: (a) contain a triazole ring and (b) were extracted *exclusively* from the claims or description sections of granted US patents published in 2014, which, in turn, (c) have a specific combination of relevant international patent classification (IPC) codes (e.g. the code ‘C07D’ stands for ‘heterocyclic organic chemistry’, while the code A61P stands for ‘specific therapeutic activity of chemical compounds or medicinal preparations’, http://www.wipo.int/classifications/ipc/en), and finally, (d) they mention the term ‘diabetes’ in their title or abstract (Supplementary Figure S1). The advantage of combining chemistry searches with keyword and metadata queries is that, perhaps for the first time in a public resource, a user can access and retrieve the relevant patented chemical space associated with a particular disease or indication, as reported in the relevant patent literature.

**Figure 4.**
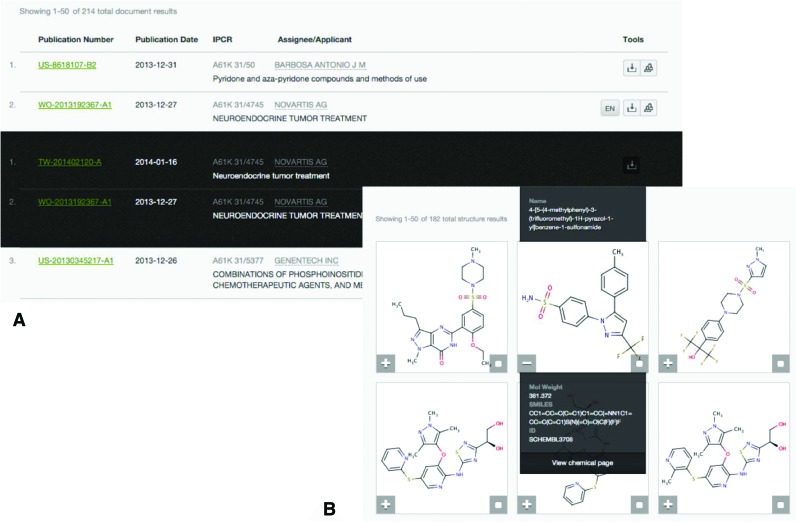
The results of search can be either a list of documents (**A**) or compounds (**B**). The former is sorted in reverse chronological order and provides a preview of the each document by means of patent ID, publication date, assignee, classification code(s), title and language. Moreover, for each document, members of the same patent family (i.e. a number of patent documents by the same inventors describing the same invention filed in multiple countries) across different patent authorities may be retrieved (listed in dark background). Finally, the chemistry annotated in each document can be exported and downloaded. In case of the compound hits (B), the report card view may be viewed for each hit (e.g. https://www.surechembl.org/chemical/SCHEMBL16354556 and Supplementary Figure S2). Additionally, users may choose a number of these search hits and retrieve the patent documents associated with their selection.

**Figure 5.**
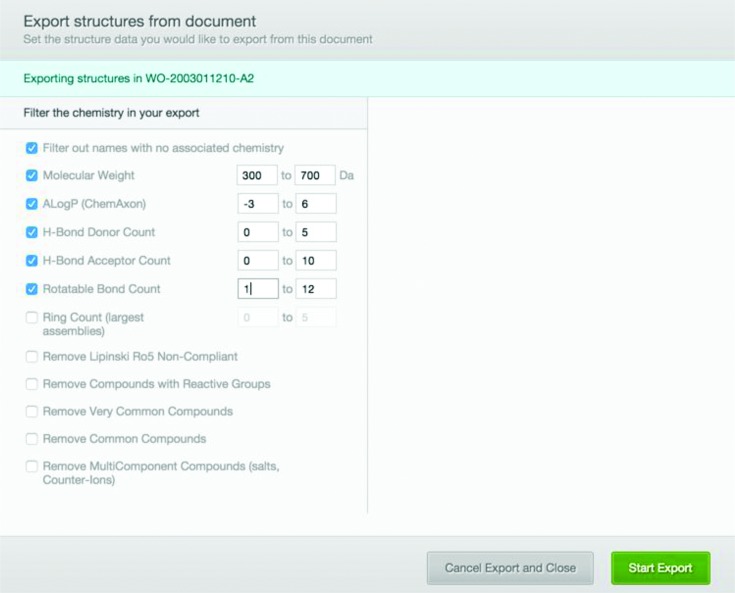
The export chemistry modal window allows users to filter compounds based on calculated physicochemical and related properties, simple counts and frequency of occurrence.

Having run a keyword-based search, users are presented with a list of patent document hits, sorted in reverse chronological order (Figure 4A); the full text of a patent document of interest may then be viewed. This view also provides the annotations of the chemical entities found in text or images highlighted in light blue, if the conversion to structure was successful, or in grey, if the conversion failed. Clicking on an annotation reveals the corresponding structure(s) along with its unique SCHEMBL ID and a link to the compound report card (Supplementary Figures S2 and S3). Furthermore, the full text view features the chemistry exporting functionality, which allows users to download a subset or the entirety of the compounds annotated in the particular patent in xml and csv format. Importantly, the interface provides a compound filtering mechanism based on (i) desired physicochemical property ranges and simple counts (e.g. molecular weight, calculated logP and number of rings), (ii) derived properties such as the Lipinski rule of five compliance (26) and finally text-mining properties such as the global frequency count, defined as the number of times a particular compound occurs in the entirety of the SureChEMBL annotated corpus, which is linked directly with the compound's novelty (Figure 5). The filtering mechanism enables users to easily remove undesired and trivial chemistry typically mentioned in a patent, such as solvents, reagents, substituents and radicals and focus on the chemical space of interest.

In the case of a chemistry-based or combined chemistry and keyword-based search, a list of compound hits is retrieved instead (Figure 4B). Users may then choose a number of the hits and retrieve the list of documents these are found in colour-coded by compound, subject to any additional query filters specified in the original search. From the document list, users may navigate to the full text view and export the chemistry, as described in the previous section.

It is also worth mentioning that help is provided for every feature and functionality marked by a question mark on the interface. All help articles along with a user support form are hosted in the accompanying knowledge base portal at https://www.surechembl.org/knowledgebase/.

### Downloads

In addition to the SureChEMBL interface which accommodates many common use-cases, the complete set of compound structures extracted from text, images and mol files, as part of the SureChEMBL pipeline, is also freely available to download from the ChEMBL ftp server as a structured-data (SD) or a tab-separated file. This allows users to query the data locally and integrate the compounds with their own chemical structure repositories. It also enables large-scale chemoinformatics analyses of chemical space, scaffolds and rings for compounds claimed in patents. The data dump consists of the back file of compounds along with incremental updates with the new compounds extracted from new patent documents, which are added to the ftp site on a quarterly basis (ftp://ftp.ebi.ac.uk/pub/databases/chembl/SureChEMBL/data/).

In order to enable bulk storage and access of SureChEMBL data, a second set of files is available to download. These provide a comprehensive compound-patent association map, i.e. they include information indicating the compounds extracted from a specific section of a specific patent document. The files provide information on the compound (SCHEMBL ID, SMILES, standard InChI Key, global corpus frequency), patent (ID and publication date) and their association (patent section and frequency of occurrence within that section). The map files allow users to integrate and analyse SureChEMBL data in their own local environments. In line with the compound dump files, the map files consist of a back file including patents published before 1 January 2015, along with incremental updates with the new associations between compounds and new patent documents, which are added to the ftp site on a quarterly basis.

All files are provided under a Creative Commons Attribution-ShareAlike 3.0 Unported license (http://creativecommons.org/licenses/by-sa/3.0).

### Data client

An alternative way to access the SureChEMBL data in bulk is the data client (https://github.com/j-siddle/surechembl-data-client). This is a data-loading Python script that firstly creates a relational database (Oracle and MySQL are supported) in the user's environment and then populates the database with SureChEMBL data it retrieves from a private ftp server on a nightly basis. The data include compounds (structure and properties) and patent documents (title, publication date and classification codes), along with their association. In addition to the front file (the patent and compound information that arrives daily), the script can also retrieve the back file for each past year and backfill the database accordingly. In the end, the data client script effectively creates a local and live snapshot of the SureChEMBL database, which allows for further integration, querying, filtering, deployment, etc., on the user's side. For example, users can install a chemical cartridge to enable chemistry searches, provide web service access and briefly replicate a large part of the functionality provided by the main SureChEMBL interface. Compared to the map files, the data client provides more information in a more timely fashion (daily versus quarterly) at the cost of additional effort and resources spent for its configuration.
